# Autoimmune aspects of Alzheimer's disease as exemplified by the diversity of autoantibodies found in patient serum and CSF

**DOI:** 10.1002/alz70856_096490

**Published:** 2025-12-24

**Authors:** Miyo K Chatanaka, Colin L. Masters, Eleftherios P Diamandis

**Affiliations:** ^1^ University of Toronto, Toronto, ON, Canada; ^2^ The Florey Institute of Neuroscience and Mental Health, Melbourne, VIC, Australia; ^3^ Sinai Health System, Toronto, ON, Canada

## Abstract

**Background:**

The current evidence supporting the complex and multifaceted etiology of Alzheimer's disease (AD) grows by the day, and it has prompted increased research in non‐“amyloid hypothesis”‐related pathways. One of these pathways of interest pertains to an autoimmune component in this disease.

**Method:**

We systematically compiled published literature supporting the view of autoimmunity in AD between 1988 and 2024, sourced from PubMed. This review critically discusses the current evidence of potential contributors to autoimmunity to AD pathobiology and describes the putative role of autoantibodies detected in patient biofluid. Special consideration was given to evaluating whether the reported autoantibodies represent true or false discoveries and the integrity of the methodological techniques.

**Result:**

The majority of reported putative AD‐related autoantibodies differ dramatically between published studies, raising doubts about the reliability and robustness of these findings. Particularly, autoantibodies may be found in a percentage of patients from the same cohort, but the same autoantibodies are undetectable in cohorts of different patients.

**Conclusion:**

Despite consistent identification of some autoantibodies in AD, their potential causal role in disease initiation and progression has not been experimentally demonstrated. We suggest follow‐up confirmatory and validation studies with sufficient power, preferably by employing orthogonal testing techniques. Uncovering the putative autoimmune components of AD is important and could pave the way to new concepts for AD pathogenesis, diagnosis and therapy.

